# Multipath Routing in Wireless Body Area Sensor Network for Healthcare Monitoring

**DOI:** 10.3390/healthcare10112297

**Published:** 2022-11-17

**Authors:** Shuja Akbar, Muhammad Mohsin Mehdi, M. Hasan Jamal, Imran Raza, Syed Asad Hussain, Jose Breñosa, Julio César Martínez Espinosa, Alina Eugenia Pascual Barrera, Imran Ashraf

**Affiliations:** 1 Department of Computer Science, COMSATS University Islamabad, Lahore Campus, Lahore 54000, Pakistan; 2 Higher Polytechnic School, Universidad Europea del Atlántico, Isabel Torres 21, 39011 Santander, Spain; 3 Department of Project Management, Universidad Internacional Iberoamericana, Arecibo, PR 00613, USA; 4 Universidade Internacional do Cuanza, Cuito-Bié 250, Angola; 5 Department of Project Management, Universidad Internacional Iberoamericana, Campeche 24560, Mexico; 6 Fundación Universitaria Internacional de Colombia, Bogotá 111311, Colombia; 7 Department of Information and Communication Engineering, Yeungnam University, Gyeongsan 38541, Republic of Korea

**Keywords:** healthcare monitoring, wireless body area networks, multipath routing, energy-efficient routing

## Abstract

Mobility and low energy consumption are considered the main requirements for wireless body area sensor networks (WBASN) used in healthcare monitoring systems (HMS). In HMS, battery-powered sensor nodes with limited energy are used to obtain vital statistics about the body. Hence, energy-efficient schemes are desired to maintain long-term and steady connectivity of the sensor nodes. A sheer amount of energy is consumed in activities such as idle listening, excessive transmission and reception of control messages, packet collisions and retransmission of packets, and poor path selection, that may lead to more energy consumption. A combination of adaptive scheduling with an energy-efficient protocol can help select an appropriate path at a suitable time to minimize the control overhead, energy consumption, packet collision, and excessive idle listening. This paper proposes a region-based energy-efficient multipath routing (REMR) approach that divides the entire sensor network into clusters with preferably multiple candidates to represent each cluster. The cluster representatives (CRs) route packets through various clusters. For routing, the energy requirement of each route is considered, and the path with minimum energy requirements is selected. Similarly, end-to-end delay, higher throughput, and packet-delivery ratio are considered for packet routing.

## 1. Introduction

Wireless body area network (WBAN) has significantly improved remote healthcare by providing continuous monitoring with wearable or cloth-embedded transducers and implementable body sensors. HMS increases the detection of emergency conditions to improve quality of life by allowing patients to not be limited to the bed and to move around within a specific distance beside a monitor or base station. A base station is a resourceful machine with dedicated energy resources, whereas the rest of the sensor nodes have limited battery backup. A sensor node expends most of its energy in communication compared to energy consumption in sensing, thus intermittent unavailability of one or more sensors can compromise the reliability of the system, resulting in unrecorded medical emergencies. So, systems based on WBAN require energy-efficient routing protocols for reducing energy consumption, ensuring reliable remote monitoring, and for the distribution of the processing load as well as network transmission. Avoiding unnecessary transmission and introducing compression may significantly improve the performance of the network as well as battery usage.

The existing solutions improve WBAN availability by enhancing the sensor battery due to the employment of various techniques, such as clustering, optimizing frame length, SDN-based routing, aggregated routing, and energy-aware routing [1,2,3,4,5,6]. However, enhancing the coverage area of WBAN while preserving the battery needs an optimal approach, such as multi-hop routing, that uses hop-to-hop relay, thus avoiding the requirement of excessive transmission power.

The proposed solution provides network optimization and distributes the processing tasks to keep nodes alive in the network, even with very few resources. To achieve network optimization, a multi-hop transmission of data is introduced to reduce the use of energy while maintaining the signal strength to transmit data over a long distance. A neighboring node with an acceptable batter level may take the responsibility of transmitting data on the behalf of a sensor having limited energy resources. For processing, an algorithm is proposed to assign data-aggregation tasks to the nodes selected as head nodes after considering numerous factors, such as remaining battery power, processing capability, and distance from the base station. The main contributions of this paper are as follows:

- A region-based energy-efficient multipath routing approach is presented that divides the entire network into clusters to increase the lifetime of sensors in HMS.
- A mechanism to select cluster representatives is proposed to route the packets through different clusters after selecting a path requiring minimum energy.
- The proposed scheme provides better delay, throughput, packet-delivery ratio, and energy consumption as compared to other schemes.

This paper is structured as follows. Section 2 contains the discussion of important works related to this study followed by the proposed methodology and region-based energy-efficient multipath routing algorithm in Section 3. Section 4 provides a performance analysis of simulation results. Lastly, the conclusion is given in Section 5.

## 2. Literature Review

This section contains a discussion of important works related to energy-efficient solutions in wireless body area sensor networks (WBASN) for healthcare monitoring. The existing work is evaluated against different comparison parameters, such as end-to-end delay, throughput, packet-delivery ratio, and approaches used to prolong the network lifetime of medical sensors. The proposed approaches are weighed based on routing strategies for energy-efficient WBASN, identifying the need for a multipath routing protocol.

In [1], an energy-efficient clustering mechanism is proposed to extend the battery life of medical implants by forming clusters in immediate proximity. The implementation is based on a modified low-energy adaptive clustering hierarchy (LEACH), and the results are compared with pure ALOHA. The data of non-critical events are aggregated, whereas, in case of an emergency, the implant data are immediately transmitted to the sink node. The evaluation providing a comparison with a trivial medium access control protocol lacks the routing parameters imperative to justify the novelty of the proposed scheme. Authors in [2] proposed an optimization method to address poor channel conditions leading to low network utility and delivery probability. The method quantitatively analyzes the relationship between the factors, which reduces the waiting delay and sets the data frame length considering the channel model. The method achieves comparable performance in terms of network utility, frame delivery probability, delay, and energy efficiency. Further improvement in QoS and energy efficiency needs the incorporation and evaluation of various routing strategies.

N. Samarji et al. [3] presented an ERQTM scheme realizing an energy-efficient routing protocol and QoS traffic management mechanism, aiming to elongate the network lifetime, ensuring network reliability. The energy-efficient routing selects an optimal cluster head considering different QoS metrics, such as nodes’ residual energies, energy consumption rates, distance to a controller, and path loss effect, to define the objective function genetic algorithm. The proposed scheme also cuts off the routing delay by prioritizing the emergency data with a high transmission rate. The proposed scheme is compared with the other priority-based scheme to demonstrate better network stability, network lifetime, residual energy, throughput, and end-to-end delay.

A robust optimization algorithm is proposed in [7] to save the power of energy-constrained sensors by using a generalized gamma distribution considering outage probability and packet retransmission. The authors proposed an alternative optimization approach to determine the solution, formulating an optimization problem as semi-strictly quasi-convex in each decision variable, at reduced complexity. The result evaluation concludes that transmission in smaller packets is preferable to avoid the retransmission request, thus achieving 47% more energy efficiency. Laya et al. [8] developed an energy-efficient data aggregation technique for WBAN using bee swarm optimization that optimizes energy efficiency by reducing the retransmission and controlling the packet congestion which ultimately results in improved energy efficiency. The bee swarm optimization approach is used to choose appropriate cluster heads from the randomly chosen cluster heads. The cluster formation step ensures that an equal and small number of nodes is allowed in each cluster to allow transmission from a member node to the sink node with less energy.

Hung et al. [9] presented an energy-efficient cooperative routing scheme for heterogeneous sensor networks, where a sensor relays a packet of not only its wireless sensor networks (WSN), but also the packets of other WSNs by dynamically established routing paths based on the residual energy, underlying sensors, and their neighbors. The proposed scheme is compared with DEEC, M1-LEACH, and M2-LEACH, showing significant improvement in the lifetime of sensors. K. Fathima et al. [10] investigated different hierarchical energy-efficient routing protocols for performance analysis of the various algorithms, such as fuzzy logic-network coding energy-efficient (FL-NC-EE) and energy-efficient clustering protocols, such as LEACH, LEACH-FL, K means-LEACH and FL-EE/D. The parameters considered for the evaluations include throughput, average lifetime, dead nodes per round, alive nodes per round, FND, QND, 10PND, etc. The authors concluded that the FL-EE-NC protocol outperforms other algorithms and techniques considering metrics of network lifetime and energy consumption efficiency.

HajilooVakile et al. [11] introduced a method to improve energy consumption in WBAN. The energy consumption problem is addressed by reducing the data size by introducing an effective data compression technique. A modified Huffman method was implemented on medical data that saves 11.8% more energy compared to NIS. Authors in [12] proposed an energy-efficient transmission power control for a wireless body area network dealing with energy losses due to constant transmission power that becomes ineffective due to large temporal changes. The solution works as an energy-efficient adaptive power control algorithm that adaptively adjusts the transmission power based on feedback from the base station. However, energy efficiency is achieved at the cost of the packet loss ratio that affects link reliability in the network.

A reliable and energy-efficient cooperative routing algorithm for wireless monitoring systems is proposed in [13]. The proposed algorithm was introduced to guarantee a performance trade-off between reliability and energy efficiency. To limit the amount of energy spent, the algorithm performs actions based on samples. The proposed solution is based on a modified version of the AODV routing algorithm that works in two phases, route discovery and route maintenance. Zhao et al. [14] presented an energy-efficient region-based routing protocol for low-power lossy networks (RPL). The proposed energy-efficient and self-region-based RPL reduces energy consumption while propagating the whole network to discover a reliable P2P route. The proposed solution achieves energy-efficient data delivery without compromising link reliability. The proposed scheme uses only a subset of nodes instead of all nodes required for route discovery.

A cooperative transmission strategy for body-area networks (BANs) in HMS is presented in [15] that offers energy saving via applying cooperative communication. Two strategies were devised, namely, direct and relay communication. In the direct cooperative strategy, two wearable devices (sensors) operate as cooperative multiple input–multiple output (MIMO) while in the relay cooperative strategy, implantable and wearable devices work together by using MIMO and these save energy during data transmission in BANs.

Routing protocols such as RPL takes either energy or reliability metric into account. Chang et al. [16] presented an energy-efficient oriented routing algorithm in WSNs that is based on the rank (approximating the node’s distance) and the objective function (how RPL selects and optimizes routes). It improves the RPL routing protocol by counting the expected transmission count (ETX) and remaining energy metrics. The link qualities are considered in ETX such as latency and packet loss that may significantly influence network connectivity. Authors in [17] have proposed medium access control for WBAN with QoS provisioning and energy-efficient design. The proposed solution works in the area of tracking and maintenance of WBAN for the quality of services, such as delivery probability and latency. The proposed solution works as a MAC protocol that ensures energy efficiency in WBAN. It works as a dynamic TDMA-based protocol where the slots are allocated on the bases of channel status and application context. The solution tackles some major issues, such as collision control, idle listening, and overheating, that cause excessive use of energy resources.

An energy-efficient routing algorithm for patient monitoring in body sensor networks is proposed in [18]. The solution works as a routing algorithm for the efficient and reliable transmission of data from on-body sensors to medical personnel via multi-hop routing, which is critical for continuous health monitoring. The solution works as an energy-efficient routing algorithm by taking it as a multi-objective optimization problem (conflicting objectives combined using a weight vector). The solution obtains routes that characterize the trade-off between energy consumption and throughput. The objective of the proposed scheme is to find routes with minimum energy consumption and maximum throughput at the trade-off of end-to-end latency.

Khianjoom et al. [19] proposed anycast Q-routing in WSNs for healthcare monitoring to overcome high network traffic and to reduce large path search delays of multi-hop Zigbee-based systems. The solution works efficiently to route information to the nearest sink, as it takes only local information at each node to reduce computational, storage, and learning overheads. However, the solution works with an assumption of no energy constraint on the sink nodes. The solution takes each node as an independent learning and routing capability for making routing decisions. The anycast Q-routing in WSNs used a greedy approach for the routing decision.

An energy-efficient solution for WBAN-based HMS is proposed in [20] to deal with energy losses due to packet collision, over-emitting, over-hearing, and idle listening. The solution uses a base station (WBAN coordinator) as a sink for the WBAN nodes using Zigbee to collect information, such as the electroencephalogram (EEG), blood pressure, glucose level, and pulse rate of the human body. The solution avoids energy consumption in sleep mode; therefore, overall energy consumption is minimized.

Wan et al. [21] presented cloud-enabled wireless body area networks for pervasive healthcare that significantly enhanced the deployment of pervasive healthcare applications. The proposed solution presents an integration of WBANs with mobile cloud computing (MCC). This solution takes advantage of dynamic provisioning, scalability, and ease of integration. The conceptual architecture used introduces mobile devices to render data collected from WBAN nodes to access points and base stations.

The mobility enhancement of patients’ body monitoring based on WBAN with multipath routing is presented in [22]. Supporting mobility is one of the basic needs in the HMS. The presence of small sensors with limited energy leads to a limited sending range and a lack of support for the patient’s mobility. The proposed solution presents an ad hoc mode to establish a network and a multi-path routing algorithm. The routing algorithm works in three phases: route discovery, route maintenance, and data transmission. The route discovery is initiated on demand when a node wishes to find a way to the destination through network flooding. The route maintenance phase starts after the route discovery as with the mobility of the patient, the quality of the routing paths may vary. To overcome this issue, a measurement packet is sent by the destination node to the source, traversing all the intermediate nodes. The strength factor is maintained by each node to keep track of the link quality. On the other hand, the source node always tries to send data via two routes simultaneously to maximize successful packet delivery.

The literature review clearly reflects that existing schemes are focused on energy conservation mechanisms without considering the inevitable factor of routing protocols. The traditional unicast routing protocols, for mobility support, such as AODV and DSDV, also need improvements considering the special needs of WBASN. The multipath routing protocol supporting mobility has great potential to be adapted for WBASN.

## 3. Proposed Methodology

### 3.1. Design of Region-Based Energy Efficient Multipath Routing Algorithm

**Figure 1:**
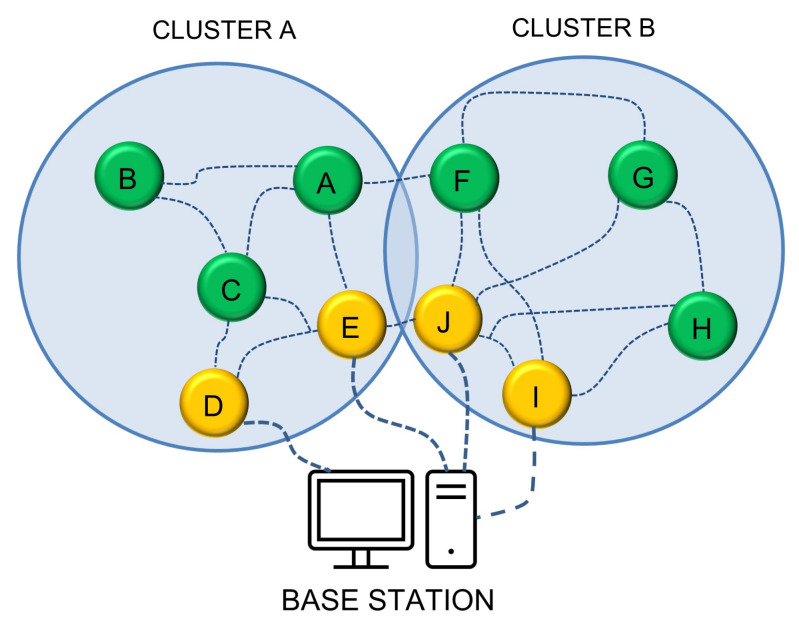
System architecture for region-based energy-efficient multipath routing.

Figure 1 presents the design artifacts of the region-based energy-efficient multipath routing approach (REMR) that provides low-energy, trustable, and efficient routing in WBAN for healthcare monitoring. In REMR, the WBASN nodes are divided into clusters and preferably multiple nodes are selected as CRs in each cluster which helps in routing packets through various clusters by the selection of a path with the least energy requirement. Algorithm 1 shows how the clusters are formed based on energy level and least distance from BS. Nodes D and E are the candidate gateway for cluster A, while the I and J nodes represent cluster B. If F needs to send data to the base station, it has an alternate path using J, E, and I nodes. The source node F has paths from its region, while it has another alternative of sending data using cluster A’s candidate node E.

REMR works in three phases: route discovery, route maintenance, and data transmission. Initially, when node F needs to transmit data to BS, it finds no direct path. It floods route requests to its neighbors J and I, and obtains responses, being the candidates. While node E via A also offers its service from the other region. F preferably selects J and I nodes and uses both paths to send data. A packet-wise approach is used in scheduling for transmitting data to the base station.

The CRs are selected by the base station (BS) based on their distance from the BS. The BS also selects multiple nodes to serve as intermediate clusters with no direct access to BS. Clusters near BS will route traffic using multiple paths and multiple CRs, resulting in decreased energy consumption for network propagation, route discovery, and control messages. We assume that the BS does not have any buffer constraints. For packet routing, the selection of a cluster candidate (CC), from the available CRs, is made based on its energy level (battery backup measured in joules) and its distance to the next hop. The selection criteria of CC are given in Equation (1), where $E_{i}$ is the energy level of node *i* and $T^{{}^{\prime }}$ is the threshold value.

(1) $$\left\{\begin{array}{c} E_{i}>T^{\prime }\\ \Delta d=MIN(\Delta x,\Delta y,\Delta z,\ldots ,\Delta n) \end{array}\right.$$

**Algorithm 1** Cluster formation based on energy level and shortest distance**Input:** Set of WBASN nodes with their energy levels and coordinates
**Output:** Set of clusters of WBASN nodes (S)
  1: **Begin:**
  2: Let Routing Table (RT) of WBASN node be *ϕ*
  3: Let R’ be the range of BS
  4: **for** each cluster $C_{i}$ **do**
  5:     **for** each WBASN node $S_{i}$ **do**
  6:         hopCount = closest neighbor hop
  7:         minDist = strongest wireless range
  8:         **for** each WBASN node $S_{j}$ in cluster $C_{i}$ where i≠j **do**
  9:            Get total resource value $r_{i}$
10:            // Calculate 1-hop neighbors based on Range R’
11:            **if** $S_{j}$ in Range of BS and hopCount = 1 and minDist = TRUE **then**
12:                // if *S_i_* is neighbor node or at 1–hop count
13:                Assign $S_{j}$ as $C_{i}$
14:            **end if**
15:        **end for**
16:        Update RT
17:    **end for**
18: **end for**
      **return**
*S*
      **end:**


A CR cannot be a CC if its energy level is less than the defined threshold value. This is to ensure that the node has the required energy to stay alive and transmit data. A CC must lay in the shortest path from the source node to the destination, while it should also be the node nearest to the source node. The selection of such a candidate will ultimately require less emitting power to generate signals, and hence, improved energy utilization will be achieved. Additionally, for a CR to be selected as CC, it must be the nearest node to the next hop having a distance Δd to the source.

Using multiple CRs serves as a cluster to become connected with BS with alternative paths available. Such candidates can help reduce the required transmission power due to the least transmission power requirements. The multipath solution also reduces the load on a single node or link; hence, link reliability is improved. Multipath scheduling also avoids the collision of packets, reducing retransmission requirements and the generation of excessive control messages. REMR is a reactive protocol, and only region-based propagation is needed. BS selects CRs on the bases of their distance to each node, and it picks multiple nodes to serve as representatives of different intermediate clusters with no direct access to the base station. Clusters near BS will route traffic through multiple paths CRs. The proposed solution decreases the energy consumption in network propagation, and route discovery, and avoids excessive control messages.

Another rather important criterion for the selection of CR is based on the signal strength of the RFID. Once the CR is selected based on the shortest distance to BS and energy value, that particular CR selects the RFID device/identifier that is installed nearest to it. In a healthcare scenario, where there is a requirement for more than one RFID device/identifier, the identifications of the tags/sensors/nodes are based on uniform distribution. This is performed to keep this clustering of sensors/tags based on RFID devices region at a secondary level. The primary clustering will be based on the received signal strength indication (RSSI) of the CR. The RFID identifier grabs the information of every tag and stores it in its memory. The RFID identifier stores the tag information through the reading order function r = {EPC, Reader ID, time}, where EPC is the electronic product code of the tag/sensor. Each identifier creates its unique key for the tags under its radar. The unique key helps in the identification of the tag by other RFID identifiers in case the tag/sensor is mobile. Each RFID identifier has *t* number of tags. Our algorithm assigns a unique ID to each RFID identifier. This unique ID is shared with all the tags of that particular RFID identifier. Suppose a sensor/tag $S_{t}$ belongs to the RFID identifier $R_{i}$, then $K_{Ri}$ is the ID of $R_{i}$ stored in $S_{t}$. $S_{t}$ needs to have this information to identify the region so that once $S_{t}$ comes back to the same region, it does not have to restore the same information. The same goes for the $R_{i}$ when it has to identify $S_{t}$ again. The RFID identifier is connected to BS. The BS receives all the secret information by the RFID identifier regarding tags during the deployment of the algorithm. The RFID identifier issues a set of secret information to BS for each RFID identifier of the system. The set of secret keys along mapping to respective tags is denoted by *z* = {($K_{Ri}$,$\Sigma_{i}$) | 1≤i≤t}, where $K_{Ri}$ is the secret key of the RFID identifier and $\Sigma_{i}$ is the mapping of the identifiers of the group $\beta_{i}$ with the secret keys of the tags, and *t* is the number of tags.

Now there are two regions also shown in Figure 1. The rectangular region $R_{e}\left(x,y\right)$, which is the RFID identifier and the circular region $C_{r}\left(x,y\right)$, where *x* is the location of the tag/sensor, and *y* is the probability of the range *x*. To ensure the security and energy efficiency of the system, our algorithm makes sure each RFID identifier communicates only with the CR besides sharing some secret ID information. It is the job of the CR to let other tags/sensors/nodes know about the reliability of the information. The CR through secret ID information identifies and trusts the information coming from the RFID identifier, and the RFID identifier has its own algorithm to verify the certainty of the data. Among both regions $R_{e}\left(x,y\right)$ and $C_{r}\left(x,y\right)$, the priority by the algorithm for ensuring energy efficiency and reliability of the information is given to the $C_{r}\left(x,y\right)$ because $C_{r}\left(x,y\right)$ interacts with more tags/sensors and makes a trustable relation and energy-efficient routing. The RFID identifiers are usually backed up with power backups. Our algorithm relies on probabilistic minimalism ($P_{m}$) in selecting a reliable region. There is a very slight chance where priority is given to $R_{e}\left(x,y\right)$ just because when $C_{r}\left(x,y\right)$ has all new tags/sensors and CR has no information about them. The relation between $R_{e}$ and $C_{r}$ by probabilistic minimalism is given by an integrated function over both regions and coordinates of nodes in them as shown by Equation (2).

(2) Pm=∫itfRexi,yi,Crxi,yigxi,yijdx

Here, *i* denotes the tags/nodes and identifiers, and g(x,y) is a function that returns all the locations of all tags and identifiers. The function *f* in Equation (2) shows how the most trusted values are extracted from both regions, each being donated by its coordinates and the number of keys stored in it. This is further elaborated by expanding *f* as follows:

(3) $$P_{m}=\;\int_{i,j}^{t}\sum \frac{\sum_{u,=1}^{w}l(K_{Ru\in St\;}⋂K_{Su\in Ri}),\;l(K_{CRu}⋂K_{Ru})\;}{\varphi (R_{u},\;S_{u},{CR}_{u})}$$

Equation (3) shows the summation of the list of secret identifiers *K* of all the tags governed by the RFID identifier and the list of all the RFID identifiers governed by all tags sorted out by their respective owners, including the CR. This integrated summation shows the probability of the successful region which contains most of the keys showing which part of the network is most trustable.

All the functions discussed in Equation (3) are explained in the following algorithms. Algorithm 2 selects a list of secret identifiers from either $R_{e}\left(x,y\right)$ or $C_{r}\left(x,y\right)$. Algorithm 3 selects the strongest tags/nodes among normal nodes/tags and CR. Algorithm 4 identifies the most trustable amongst the normal nodes/tags, CR, and RFID identifiers.
**Algorithm 2** Selecting list of secret identifiers**Input:** Set of RFID Identifiers
**Output:** Set of Secret RFID Identifiers K
  1: **Begin:**
  2: **for** each tag ID from u to w **do**
  3:     Select secret ID K from all the tags set by RFID identifier
  4:     Populate array from K(tags)
  5:     Select secret ID K from all the RFID identifiers set by tags
  6:     Populate array from K(ID)
  7: **end for**
  8: **if** K(tags) is bigger and old than K(ID) **then**
  9:     Sum all K(tags)
10: **else**
11:     Sum all K(ID)
12: **end if**
      **return** K **end:**


**Algorithm 3** Selecting strong tags/nodes**Input:** Set of CRs
**Output:** Set of strong tags/nodes K
  1: **Begin:**
  2: **for** each tag ID from u to w **do**
  3:     Select secret ID K from all the tags set by CR
  4:     Populate array from K(tags)
  5:     Select secret ID K from all the CRs set by tags
  6:     Populate array from K(CR)
  7: **end for**
  8: **if** K(tags) is bigger and old than K(CR) **then**
  9:     Sum all K(tags)
10: **else**
11:     Sum all K(CR)
12: **end if**
      **return**
*K*
**end:**


**Algorithm 4** Selecting most trustable set**Input:** Set of RFID Identifiers, Sets of CRs, Sets of Normal Tags
**Output:** Most trustable set
  1: **Begin:**
  2: **for** each Input Set $s_{i}$ **do**
  3:     Check combined earliest dates combinations and tag the winner with w
  4:     Check the number of entries and tag the winner with w
  5:     Check the size of the Object array and tag the winner with w
  6: **end for**
      **return** Set with most w count as the most trustable set
      **end:**


### 3.2. Tracking in x, y Coordinates

Tracking tags/nodes by the RFID identifiers is a tricky task. REMR uses the Bayes rule to infer the locations of the tags. The tags’ location is described using variable *x* and an observation variable assisting coordinate variable *y*. The joint probability distribution q(x,y) is computed over both *x* and *y*. In addition, given an observed value y, this joint probability distribution induces the computation of a conditional distribution q(x|y), which can be used to predict the object’s location. More precisely, based on Bayes’ rule, the conditional distribution q(x|y) can be represented as

(4) $$f\left(x|y\right)=\;\frac{f\left(y|x\right)f\left(x\right)}{\int f\left(y|x\right)f\left(x\right)dx}$$

Supposing we have n observations $y^{n}=\left(y_{1},y_{2},\ldots ,y_{n}\right)$ of RFID data at each time-step T(x), we replace f(x|y) with

(5) $$f\left(y_{1},y_{2},\ldots ,y_{n}|x\right)=\;\prod f\left(y_{i}\right)=T\left(x\right)$$

Thus, the current state is defined by the posterior density over the random variable location vector *x* conditioned on all RFID data:

(6) $$f\left(x|y^{n}\right)=\;\frac{f\left(y^{n}|x\right)f\left(x\right)}{\int f\left(y^{n}\right)f\left(x\right)dx}=\frac{T\left(x\right)f\left(x\right)}{h_{n}}$$

where

(7) $$h_{n}=\;\int T\left(x\right)f\left(x\right)dx\;$$

is the normalizing constant. In this case, one location variable in $X=(x_{1},\ldots ,x_{k})$, e.g., $x_{1}$, is determined by computing the following marginal posterior density:

(8) $$f\left(x_{1}|Y^{n}\right)=\;\int \int \ldots \int f\left(x_{1},\;\ldots ,\;x_{k}|Y^{n}\right)dx_{2}\ldots dx_{k}$$

These sampling points from function *f* in the form of *x* and *y* show marginal positions of the tags/nodes in the RFID region and are compared with RFID identification memory data for confirmation.

## 4. Results and Discussion

### 4.1. Experimental Setup

To test the proposed REMR, we conducted several trials in NS3 using a clustered approach. For this, we defined two clusters in a wireless scenario and tested our proposed approach with ad hoc on-demand distance vector (AODV) [23] using different parameters. Table 1 outlines the simulation parameters used for the experimental setup. AODV is selected for comparative analysis, as the comparison with a well-established routing protocol helps establish the novelty of proposed region-based multipath routing to conserve energy following a better overlay approach.

### 4.2. Results

**Figure 2:**
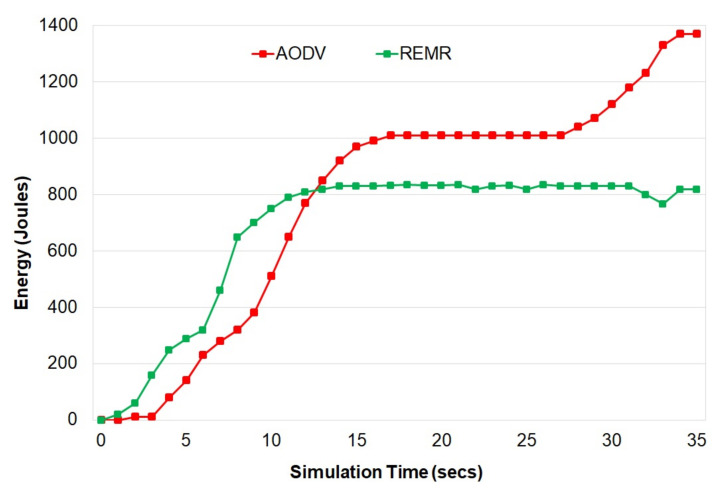
Energy consumption of AODV and REMR.

Figure 2 shows that the REMR performs better in comparison to AODV in terms of energy consumption. One thing that should be mentioned here is that at a certain point in the simulation, the energy consumed by REMR became almost constant. In comparison to that, the AODV protocol continued to consume a large amount of energy.

**Figure 3:**
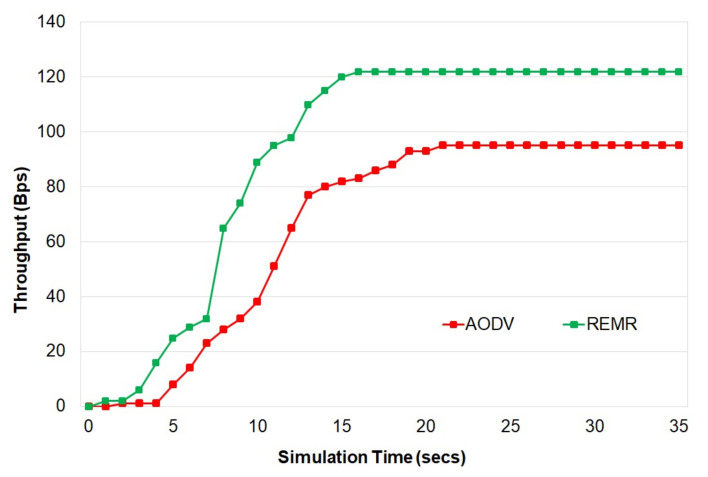
Throughput of AODV and REMR.

Figure 3 shows the results of throughput of REMR (edited AODV in the simulation environment) and AODV as implemented in the reference [20]. It is observed that REMR performs better than AODV in terms of throughput due to recursive and energy-efficient implementation in the proposed REMR. This is also because REMR uses multipath routing, whereas AODV uses single-path routing.

**Figure 4:**
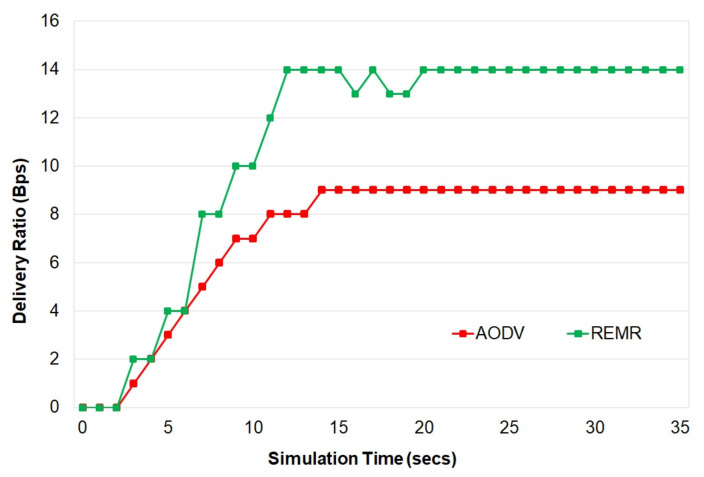
Delivery Ratio.

Figure 4 shows the delivery-to-time ratio performance of both REMR and AODV. REMR achieves a better packet delivery ratio than AODV. This is mainly due to the multipath capability of REMR.

**Figure 5:**
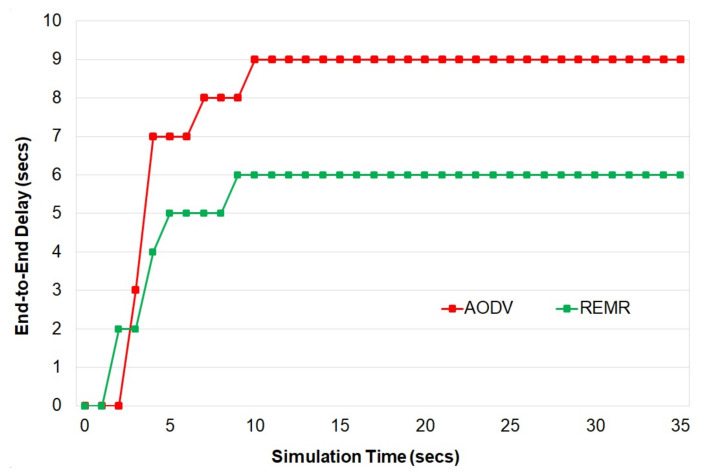
End-to-end delay.

Figure 5 represents the end-to-end delay of a particular node to the sink. REMR shows less delay in sending data from one node to the sink. Though the REMR has more end-to-end delay than the AODV at the start, after a certain amount of time, the delay starts decreasing once the better routing paths are finalized using the multipath approach.

## 5. Conclusions

Wireless body area sensor networks play a very significant role in terms of healthcare. WBASNs are used to collect the medical data of a patient linked to the HMS. However, there are some challenges regarding the transmission of data from one place to another. There is a need for a routing protocol that will be able to transmit data reliably while consuming less energy. This paper proposes a multipath routing protocol known as the region-based energy-efferent multipath routing algorithm, which, in comparison to AODV, performs better in terms of energy consumption, end-to-end delay, throughput, and packet-delivery ratio. REMR was tested in an NS3 environment using a custom two-cluster topology. After several trials, the test results were generated that prove the reliability of the REMR.

In the future, the proposed approach will be extended to include channel modeling and access control mechanisms for further improvement in results with applicability to a broader range of scenarios, such as WBASN integration with federated cloudlet and internet-of-things (IoT) architecture.

## Figures and Tables

**Table 1:** Simulation parameters.

Routing Protocol	AODV
Node Density	80
Area	6000 × 6000 (m${}^{2}$)
Node Range	100–300 m
Simulated Time	35 min
Node Type	Multiple mobile nodes with automatic harvesting
Traffic type	constant bit rate
Evaluation parameters	Throughput, energy consumption, delivery ratio, end-to-end delay
No. of Receivers	1

## Data Availability

Not applicable.
